# Dezocine and butorphanol for the prevention of sufentanil-induced cough: a randomized controlled trial investigating the impact of intravenous access site

**DOI:** 10.3389/fphar.2025.1690090

**Published:** 2026-01-07

**Authors:** Ying Liu, Hong-Yan Ma, Jing-Hui Shi

**Affiliations:** Department of Anesthesiology, The First Affiliated Hospital of Harbin Medical University, Harbin, China

**Keywords:** sufentanil-induced cough, dezocine, butorphanol, venous access, randomized controlled trial

## Abstract

**Introduction:**

Sufentanil, a potent opioid commonly used for anesthesia induction, is frequently associated with a cough reflex that may compromise airway stability and elevate intracranial or intraocular pressure. While pretreatment with opioids such as dezocine or butorphanol has been proposed to mitigate this reaction, comparative data on their efficacy are limited. This study aimed to primarily evaluate and compare the effects of dezocine and butorphanol in preventing sufentanil-induced cough (SIC), and secondarily, to investigate the impact of intravenous access site (hand vs. foot) on SIC incidence and severity during general anesthesia induction.

**Methods:**

In this randomized controlled trial, 246 ASA I–II patients (aged 18–65) scheduled for elective surgery were assigned to six groups (n = 41 each) using a random number table. Group Ia/Ib received dezocine 0.1 mg/kg via hand/foot venous access; Group IIa/IIb received butorphanol 0.02 mg/kg via hand/foot access; Group IIIa/IIIb received 5 mL normal saline via hand/foot access. All study drugs were diluted to 5 mL and administered intravenously, followed 3 min later by sufentanil 0.5 μg/kg given over 3 s. The incidence, severity (graded 1–3), and onset time (within 1 min) of SIC were recorded.

**Results:**

SIC incidence was 1.25% in the dezocine group, 0% in the butorphanol group, and 38.75% in the saline group. Both dezocine and butorphanol significantly reduced SIC incidence compared to saline (P < 0.05), with no significant difference between the two active drugs. Among saline subgroups, SIC incidence was significantly higher with hand venous access (52.5%) compared to foot access (25%) (P < 0.05), accompanied by faster onset and greater severity in the hand-access group (P < 0.05). The site of intravenous access significantly influences SIC, with foot venous access markedly reducing its incidence and severity compared to hand access. Furthermore, both dezocine and butorphanol are highly effective in suppressing SIC, with comparable efficacy between them.

**Discussion:**

These findings establish the optimization of intravenous access site as a simple, nonpharmacological strategy for SIC prophylaxis, which can be used alone or in conjunction with pharmacologic pretreatment.

**Clinical trial registration:**

https://www.chictr.org.cn/showproj.html?proj=24109, identifier, ChiCTR-IPR-17014201.

## Introduction

Sufentanil, a potent opioid analgesic, is widely used for general anesthesia induction due to its strong analgesic properties and favorable pharmacokinetics (Huang et al., 2024). However, its clinical use is often complicated by a high incidence of sufentanil-induced cough (SIC), reported in 15.0%–47.1% of cases (An et al., 2015; Agarwal et al., 2007). SIC can trigger acute airway complications such as laryngospasm or bronchospasm and may cause abrupt increases in intrathoracic pressure, potentially leading to elevated intracranial or intraocular pressure—posing serious risks for neurosurgical and ophthalmic patients (Yu et al., 2011). It can also destabilize anesthesia induction and increase the risk of aspiration and other perioperative complications (Oshima et al., 2006).

Opioid receptor-modulating agents offer promising strategies for SIC prevention (Su et al., 2016). Dezocine, a κ-receptor agonist and partial μ-receptor antagonist, is thought to exert antitussive effects by modulating μ-receptors in the medullary cough center (Xiong et al., 2020). Butorphanol, another κ-receptor agonist with weak μ-receptor antagonism, likely suppresses the vagally mediated cough reflex arc (Zhang et al., 2023). Although the antitussive properties of dezocine and butorphanol have been individually established, critical gaps persist in translating this knowledge into optimized clinical practice. First, a direct head-to-head comparison under standardized conditions is lacking, leaving clinicians without evidence-based guidance for choosing between these two effective agents. Second, and more importantly, the potential influence of intravenous access site on SIC—a simple yet fundamental procedural variable—has been entirely overlooked. It remains unknown whether administration via the hand versus foot veins, with their inherent differences in venous return circulation time, impacts the efficacy of SIC prophylaxis.

Therefore, this double-blind, randomized controlled trial was designed to address these specific gaps. It is not only directly compare the efficacy of dezocine and butorphanol but also to systematically evaluate the intravenous access site as an independent variable. Using standardized administration protocols and precise timing, this study aimed to provide a comprehensive evaluation of their effects on SIC incidence, severity, and onset. We hypothesized that the two drugs would be equally effective, and that foot venous access would confer a protective advantage. These findings offer novel evidence to guide personalized strategies for SIC prevention.

## Methods

### Study design and participants

This single-center, randomized, double-blind, controlled clinical trial was conducted to compare the preventive effects of dezocine and butorphanol pretreatment on SIC and to evaluate the impact of intravenous access site on this outcome. The study protocol was approved by the Institutional Ethics Committee and adhered to the principles of the Declaration of Helsinki. Written informed consent was obtained from all participants before surgery. The trial was carried out in the operating rooms of the First Affiliated Hospital of Harbin Medical University between October 2017 and March 2018 (Clinical Trial Registration No. ChiCTR-IPR-17014201).

Eligible patients met the following inclusion criteria: (1) age between 18 and 65 years; (2) American Society of Anesthesiologists (ASA) physical status I–II; (3) scheduled for elective surgery; and (4) voluntary participation with informed consent provided by the patient and/or their family. Exclusion criteria included: (1) chronic use of antitussives, opioids, corticosteroids, or angiotensin-converting enzyme inhibitors; (2) a history of bronchial asthma, chronic obstructive pulmonary disease, chronic cough, or upper respiratory infection within the previous 2 weeks; (3) morbid obesity; (4) hepatic or renal impairment; (5) gastroesophageal reflux disease; (6) cardiovascular conditions such as hypertension, conduction abnormalities, bradycardia, arrhythmia, or coronary artery disease; (7) intraoperative use of ephedrine or atropine; (8) endotracheal intubation lasting over 30 s or repeated intubation; (9) refusal to provide informed consent; and (10) any condition associated with elevated intracranial, intra-abdominal, or intraocular pressure.

### Randomization and blinding

Randomization was performed using a computer-generated table created by an independent statistician uninvolved in any other aspect of the trial. Patients were randomly assigned (1:1:1:1:1:1) to one of six groups (n = 41 per group):

(1) Group Ia: Dezocine 0.1 mg/kg via hand intravenous access; (2) Group Ib: Dezocine 0.1 mg/kg via foot intravenous access; (3) Group IIa: Butorphanol 0.02 mg/kg via hand intravenous access; (4) Group IIb: Butorphanol 0.02 mg/kg via foot intravenous access; (5) Group IIIa: 5 mL normal saline via hand intravenous access; (6) Group IIIb: 5 mL normal saline via foot intravenous access. Allocation results were sealed in opaque, sequentially numbered envelopes managed by a designated staff member. Envelopes were opened only after verifying eligibility and obtaining consent.

To ensure blinding, an independent pharmacist prepared all study medications. Dezocine and butorphanol were diluted with normal saline to a total volume of 5 mL. Normal saline used as placebo was undiluted. All syringes were identical in appearance and labeled with unique codes. The code key remained with the pharmacist until the study database was locked and unblinded. Anesthesiologists and researchers responsible for anesthesia management, cough monitoring, and data collection were blinded to group assignments. Predefined unblinding protocols were in place in the event of severe adverse events requiring immediate medical intervention.

### Preoperative preparation and intervention protocol

All patients refrained from preoperative medications for at least 24 h before surgery. Upon entering the operating room, an intravenous line was established using a 20G indwelling catheter (Bidi Medical Devices, Suzhou, China), inserted either on the dorsum of the hand (Groups Ia, IIa, IIIa) or foot (Groups Ib, IIb, IIIb), as per group assignment. Following IV access, 0.9% normal saline was infused at approximately 0.25 mL/s (4 drops per second), while patients rested quietly in a calm environment and received supplemental oxygen via nasal cannula at 2 L/min.

Two minutes after initiating oxygen, patients received their pretreatment medication intravenously: dezocine 0.1 mg/kg, butorphanol 0.02 mg/kg, or an equivalent volume of normal saline—all diluted to 5 mL with saline. Three minutes later, sufentanil 0.5 μg/kg was administered as a rapid IV bolus over 3 s. One minute after sufentanil injection, anesthesia was induced with midazolam 0.02 mg/kg, cisatracurium 0.2 mg/kg, and propofol 1.5 mg/kg, followed by endotracheal intubation 3 min after induction. Throughout the procedure, special attention was paid to documenting any cough occurring within 1 minute of sufentanil administration.

### Outcome definition

#### Primary endpoints

The primary outcome was the incidence of SIC, defined as the proportion of patients who developed a cough within each group.

#### Secondary endpoints

(1) SIC severity, assessed using a three-grade scale: Grade 1 (mild) = one to two coughs; Grade 2 (moderate) = 3–4 coughs; Grade 3 (severe) = ≥5 coughs or cough accompanied by apnea or thoracoabdominal muscle rigidity; (2) SIC onset time, measured from the completion of sufentanil injection to the first cough. All data were recorded in real time by the anesthesiologist into electronic case report forms and verified by independent researchers to ensure completeness and accuracy.

### Statistical analysis

Based on pilot data and literature, the SIC incidence in the saline control group was estimated at approximately 40% (He et al., 2020). Assuming the incidence in the dezocine and butorphanol groups could be reduced to below 5%, with a two-tailed alpha of 0.05 and power of 80%, the required sample size per group was calculated as 38 using PASS 15.0 software. To account for an anticipated 5% dropout rate, the final sample size was increased to 41 per group, yielding a total of 246 patients across six groups.

The study employed a 3 × 2 factorial design based on Pretreatment Drug (Dezocine, Butorphanol, Saline) and Injection Site (Hand, Foot). For the primary outcome of SIC incidence, a preliminary analysis revealed a highly sparse data distribution. This “zero-inflated” data structure precluded the use of parametric factorial models like two-way ANOVA, as the model assumptions would be violated and the estimates would be unstable. Therefore, to robustly address the primary objective of comparing drug efficacy, the data were collapsed across the Injection Site factor for the between-drug comparison. The incidence of SIC among the three combined drug groups (Dezocine, Butorphanol, Saline) was compared using the Chi-square test. For secondary outcomes where sufficient data were available (i.e., within the Saline group), the effect of Injection Site on continuous variables (e.g., onset time) was analyzed using an independent samples t-test, and on categorical variables (incidence, severity) using the Chi-square or Fisher’s exact test. This approach allowed for a valid assessment of the site effect in the absence of effective pharmacologic suppression. Normally distributed continuous variables across the three main drug groups (collapsed for site) were expressed as mean ± standard deviation and compared using one-way analysis of variance (ANOVA), with LSD-t tests for post hoc pairwise comparisons. A *P*-value < 0.05 was considered statistically significant. Subgroup analysis to evaluate the effect of IV access site was performed using stratified χ^2^ testing. All statistical analyses were conducted using SPSS version 26.0 (IBM Corp).

## Results

### Baseline characteristics

A total of 246 patients were enrolled in the study. Of these, one was excluded due to an allergic reaction, and five were excluded due to prolonged intubation time (>30 s) or multiple intubation attempts. None of the remaining patients experienced hypoxia (SpO_2_ <90%), apnea, or chest wall rigidity. Ultimately, 240 patients were randomized into six groups and included in the final analysis. Baseline characteristics are presented in Table 1. There were no statistically significant differences among the groups in ASA classification, sex, age, height, weight, or BMI (*P* > 0.05).

**TABLE 1:** Demographic data of six groups patients.

Group	Number	ASA (I/II)	Gender (male/female)	Age (years)	Height(cm)	Weight (kg)	BMI (kg/m^2^)
Ia	40	24/16	18/22	45.23 ± 11.01	165.75 ± 7.34	62.76 ± 8.37	22.83 ± 2.32
Ib	40	26/14	18/22	46.25 ± 9.70	166.70 ± 7.80	63.20 ± 7.11	22.71 ± 1.61
IIa	40	22/18	18/22	46.30 ± 9.13	166.20 ± 7.99	62.39 ± 9.21	22.5 ± 2.28
IIb	40	27/13	18/22	45.25 ± 9.71	165.55 ± 7.61	62.48 ± 7.77	22.74 ± 1.89
IIIa	40	28/12	17/23	46.88 ± 9.35	165.83 ± 8.43	61.31 ± 9.11	22.19 ± 1.80
IIIb	40	24/16	17/23	45.25 ± 8.04	165.88 ± 8.69	62.85 ± 7.22	22.81 ± 1.48

ASA, american society of anesthesiologists; BMI, body mass index.

### Comparison of SIC incidence, severity, and onset time of SIC among dezocine, butorphanol and normal saline groups

Detailed outcomes for all six subgroups are presented in Table 2. Within the saline control group, the incidence of SIC was 52.5% (21/40) in Group IIIa (hand) and 25.0% (10/40) in Group IIIb (foot). In stark contrast, the incidence in the active drug groups was markedly low. In the dezocine group, a single cough event occurred in Group Ia (hand access, 2.5%), while no cough events occurred in Group Ib (foot), Group IIa (butorphanol/hand), or Group IIb (butorphanol/foot).

**TABLE 2:** Incidence, severity, and onset time of SIC in the three groups.

Outcomes	Category	I (a+b) (n = 80)	II (a+b) (n = 80)	III (a+b) (n = 80)
Time of onset of cough (s)		20^b^	—	18.68 ± 4.50^c^
Incidence rate (%)		1 (1.25%)^a^	0 (0%)	31 (38.75%)
Severity of SIC	No	79 (98.75%)	80 (100.0%)	49 (61.25%)
	Mild	1 (1.25%)	0 (0%)	13 (16.25%)
	Moderate	0 (0%)	0 (0%)	9 (11.25%)
	Severity	0 (0%)	0 (0%)	9 (11.25%)

^a^ The single cough event occurred in the hand venous access subgroup (Group Ia);

^b^ Onset time is not applicable for meaningful statistical comparison due to the extremely low number of events (n ≤ 1).

^c^ Data are presented as mean ± SD, for the saline group only.

Given the near-complete suppression of cough in the active drug subgroups, the data were collapsed for the primary drug comparison. The overall incidence of SIC was 1.25% (1/80) in the combined dezocine group (I), 0% (0/80) in the combined butorphanol group (II), and 38.75% (31/80) in the combined saline group (III). The incidence of SIC in Group III (normal saline) was significantly higher than in Groups I (dezocine) and II (butorphanol) (*P* < 0.05). No significant difference in SIC incidence was found between Groups I and II (Table 3). Regarding the injection site effect, when data were combined across all drug groups, the overall incidence was higher in hand access groups (18.3%, 22/120) compared to foot access groups (8.3%, 10/120) (*P* < 0.05; Table 3).

**TABLE 3:** Multiple comparisons of SIC Incidence between combined groups.

Comparison	Number	ASA (I/II)
Main drug effect	Dezocine (I) vs. Saline (III)	1/80 (1.25%) vs. 31/80 (38.75%)
	Butorphanol (II) vs. Saline (III)	0/80 (0%) vs. 31/80 (38.75%)
	Dezocine (I) vs. Butorphanol (II)	1/80 (1.25%) vs. 0/80 (0%)
Main site effect	Hand (a) vs. Foot (b)	22/120 (18.3%) vs. 10/120 (8.3%)

ASA, american society of anesthesiologists.

It is noteworthy that the single cough event in the dezocine group occurred in the hand venous access subgroup (Group Ia). In terms of severity, Group III exhibited significantly more intense cough responses compared to Groups I and II (*P* < 0.05). However, due to the extremely low number of cough events in Groups I and II (n = 1 and n = 0, respectively), a meaningful statistical comparison of SIC onset time among these groups was not feasible (Table 2).

### Effect of injection site on SIC incidence, severity, and onset time in the normal saline group

To isolate the effect of intravenous access site from the dominant pharmacologic effect, we analyzed this factor within the saline control group alone. Among these patients, SIC incidence was significantly higher with hand venous access (52.5%) compared to foot access (25%) (*P* < 0.05; Table 4), accompanied by faster onset and greater severity in the hand-access group (*P* < 0.05; Table 4). This confirms that the injection site is an independent and significant predictor of SIC.

**TABLE 4:** The incidence, severity and and onset time of SIC in patients with different injection sites in saline group.

Outcomes	Category	IIIa (n = 40)	IIIb (n = 40)
Time of onset of cough (s)		17.14 ± 3.04^a^	21.9 ± 3.78^a^
Incidence rate (%)		21 (52.5%)	10 (25%)
Severity of SIC	No	19 (47.5%)	30 (75%)
	Mild	9 (22.5%)	4 (10%)
	Moderate	6 (15%)	3 (7.5%)
	Severity	6 (15%)	3 (7.5%)

^a^ Data are presented as mean ± SD, for the saline group only.

## Discussion

This randomized controlled trial was designed to address two under-explored questions in the prevention of SIC: the direct comparative efficacy of two established antitussive agents (dezocine and butorphanol), and the novel role of intravenous access site as a modifiable risk factor. Our findings confirm the comparable high efficacy of both drugs and, for the first time, demonstrate that foot venous access independently reduces the incidence and severity of SIC. The key findings were as follows: (1) pretreatment with dezocine (0.1 mg/kg) and butorphanol (0.02 mg/kg) significantly reduced SIC incidence (1.25% and 0%, respectively) compared with normal saline (38.75%); (2) there was no statistically significant difference between the two drugs in preventing SIC; and (3) among patients in the normal saline group, foot venous access was associated with lower SIC incidence (25% vs. 52.5%), reduced severity, and delayed onset time compared to hand access. These results confirm the clinical efficacy of both dezocine and butorphanol and, for the first time, highlight the independent effect of intravenous administration site on SIC. This provides a new perspective for optimizing clinical prevention strategies.

Although the precise mechanism of SIC remains unclear, several hypotheses have been proposed: (1) activation of specific receptors in the airways, such as C-fiber and opioid receptors (Karlsson et al., 1990); (2) increased airway sensitivity following tracheal stimulation (Zhang and Chen, 2006); (3) direct stimulation of airway C-fibers by sufentanil citrate (Tanaka and Maruyama, 2003; Tanaka and Maruyama, 2005); and (4) reduced chest wall compliance and glottic closure induced by sufentanil, leading to cough (Yemen, 1998; Horrow et al., 1991; Bennett et al., 1997). This study supports the view that both dezocine and butorphanol suppress SIC through opioid receptor modulation. Dezocine, a κ-receptor agonist and partial μ-receptor antagonist, likely inhibits the cough reflex by dampening excessive μ-receptor activation in the medullary cough center. Prior studies suggest that μ-agonists like sufentanil trigger coughing via μ-receptors in the nucleus tractus solitarius, and dezocine may block this pathway through its partial antagonism (Liu et al., 2014; Gui et al., 2023). Butorphanol, with dual action as a κ-agonist and weak μ-antagonist, is thought to suppress the peripheral vagus-mediated cough reflex. After sufentanil activates vagal nerve endings, butorphanol may reduce signal transmission to the central nervous system by diminishing neuronal conduction efficiency (Yin and Zhang, 2019; Aoki and Eki, 2024).

The lack of significant difference between the two drugs suggests similar clinical efficacy in suppressing SIC at the doses tested, despite their slightly different mechanisms. This offers practical flexibility: dezocine may be better suited for patients with compromised respiratory function due to its lower risk of postoperative respiratory depression (Sun et al., 2012), whereas butorphanol, with stronger sedative properties (Zh et al., 2021), may benefit patients with preoperative anxiety. The single cough event that occurred in the dezocine hand-access subgroup suggests that even with effective prophylaxis, SIC is not entirely abolished in all patients, particularly when using the faster hand venous route. However, due to the solitary nature of this event, we refrain from drawing any conclusions regarding the onset time or the mechanism of breakthrough cough.

In the normal saline group, foot venous access led to a significantly lower incidence of SIC, reduced severity, and delayed onset compared to hand access. This should be viewed as a proof-of-concept investigation that validates the intravenous access site as a significant and modifiable risk factor for SIC. The dramatic difference observed between hand and foot access provides a strong clinical foundation that warrants further exploration. While our study design confirms the association, it generates important hypotheses regarding the underlying pharmacokinetic mechanisms. Hand veins, part of the upper limb superficial venous system, return blood rapidly to the heart via the superior vena cava, enabling sufentanil to reach peak central concentrations more quickly and potentially trigger SIC by acting on the medullary cough center (Arner and Högestätt, 1987). In contrast, venous return from the foot travels a longer path via the inferior vena cava, affected by venous valves and lower limb muscle pump mechanisms, resulting in slower blood flow (Pinto et al., 2018). This may attenuate peak sufentanil concentration and delay its action, thereby reducing cough reflex activation.

The clinical implications of our findings regarding intravenous access site are multifaceted. First and foremost, for patients at highest risk from SIC (e.g., neurosurgical, ophthalmic), pharmacologic prophylaxis with dezocine or butorphanol remains the most effective and recommended strategy. However, the impact of venous access site holds significant value in broader practice. It provides a simple, non-pharmacological risk-reduction strategy in common scenarios where pretreatment is omitted, such as in cases of oversight, contraindications to additional opioids, or in low-risk procedures where prophylaxis is not routine. Furthermore, for the most vulnerable patients, a multi-modal approach combining pharmacologic pretreatment with foot venous access could be considered to maximize protection and minimize any residual risk. Finally, this finding is highly generalizable, as it represents a zero-cost intervention applicable to all patients receiving sufentanil.

### Strengths and limitations

This study had several strengths: (1) a randomized, double-blind, placebo-controlled design minimized selection bias and observer interference; (2) a key innovative aspect of this study was the incorporation of intravenous access site as an independent variable, uncovering a previously overlooked but clinically significant factor influencing SIC. This provides a simple, non-pharmacological strategy to mitigate SIC risk; (3) our study provides direct comparative evidence of dezocine and butorphanol for SIC prevention under standardized conditions, offering clinicians practical guidance for drug selection; and (4) Strictly standardized administration procedures—including a uniform sufentanil bolus over 3 seconds—and rigorous cough grading criteria enhanced result reliability.

However, some limitations must be acknowledged. First, the single-center design may limit generalizability, as institutional differences in venipuncture practices and patient populations may affect outcomes. Second, only fixed doses of dezocine and butorphanol were tested, preventing evaluation of dose-response relationships and optimal dosing. Third, plasma drug concentrations were not measured, limiting direct confirmation of the pharmacokinetic influence of venous access site. Lastly, ASA III–IV patients were excluded, so the results may not be applicable to critically ill individuals or those with significant comorbidities.

## Conclusion

This study identifies the choice of intravenous access site as a key modifiable factor for SIC prevention, with foot access significantly reducing its risk and severity. For pharmacological prophylaxis, both dezocine and butorphanol are equally effective options. We recommend considering foot venous access as a simple, cost-free strategy to mitigate SIC, particularly in settings where pharmacological pretreatment is not used, and in high-risk patients. The findings of this study directly point to two critical avenues for future research. First, pharmacokinetic studies measuring serial plasma concentrations of sufentanil following administration via hand versus foot veins are essential to directly confirm the mechanism behind the observed clinical difference. Second, the identification of the intravenous access site as a major predictor, alongside other patient factors, provides a key variable for developing and validating a clinical prediction model for SIC risk. Such a model would allow for personalized, risk-stratified prophylaxis strategies, potentially reserving pharmacologic pretreatment for the highest-risk individuals identified by the model.

## Data Availability

The raw data supporting the conclusions of this article will be made available by the authors, without undue reservation.
